# Healthy lifestyle behaviors and control of hypertension among adult hypertensive patients

**DOI:** 10.1038/s41598-018-26823-5

**Published:** 2018-05-31

**Authors:** Samaneh Akbarpour, Davood Khalili, Hojjat Zeraati, Mohammad Ali Mansournia, Azra Ramezankhani, Akbar Fotouhi

**Affiliations:** 1 Department of Epidemiology and Biostatistics, School of Public Health, Tehran University of Medical Sciences, Tehran, Iran; 2 Prevention of Metabolic Disorders Research Center, Research Institute for Endocrine Sciences, Shahid Beheshti University of Medical Sciences, Tehran, Iran

## Abstract

The aim of the present study was to evaluate the healthy lifestyle behaviors in hypertensive patients (aware, n = 1364 and not aware, n = 1213) based on 2011 national survey of risk factors of non-communicable disease (SuRFNCD) of Iran. Lifestyle score was calculated based on lifestyle behaviors, including smoking status, nutrition, physical activity status and body mass index separately for each patient. Of all aware patients, 27.79% (22.35–33.64) were adherence to the good lifestyle category. Almost the same percentage 29.24% (23.62–34.86) were observed in patients who were not aware of his/her illness. Moreover, adherence to good lifestyle is significantly higher in those who were aware without using antihypertensive medication (30.52% vs. 27.14%; p-value = 0.033). We also found that the prevalence of good lifestyle among patients with controlled hypertension is significantly higher than those who did not control his/her hypertension (32.54% vs. 27.59; p-value = 0.042). In people who were taking antihypertensive medication, adherence to healthy lifestyle did not have any significant relationship with the control of hypertension. The results of this study showed that awareness of hypertension did not improve people’s lifestyle. However, those who aware, but not using any antihypertensive medications are able to control his/her level of blood pressure better than those using medications.

## Introduction

Hypertension is not only an important risk factor for non-communicable disease, but also is a major cause of mortality and morbidity all over the world^1–4^. The prevalence and incidence of hypertension as well as isolated systolic and diastolic blood pressure is still increasing in most regions^5–7^. Developing countries, especially Asian countries, are expected to experience the highest increase of hypertension^8^; reports show that about two thirds of hypertensive patients live in developing countries^9,10^. About 6.6 million Iranians aged 25 to 65 years old are suffering from hypertension^11,12^. Findings show that about 54% of cases of stroke and 47% of cases of coronary artery disease are attributed to hypertension. The risk of developing coronary heart disease in hypertensive old Iranian was 96% in those without diabetes and 3.23 fold in those with diabetes; the same increasing risk was observed in case of developing stroke (2.24 and 3.73 fold increase, respectively)^3^. Since, hypertension is considered asymptomatic, nearly half of the patients are unaware of their disease^13^. Many researchers believe that major changes in lifestyle behaviors play an important role in the prevalence of hypertension^14,15^. Several studies showed that low level of physical activity, being overweight, malnutrition, and being smoker could be associated with increased risk for hypertension even in early adulthood prediction of hypertension during adolescence^16–19^. Researchers believe that taking antihypertensive medication as well as lifestyle modification will result in the best therapeutic effect^14^. However, some studies have shown that when hypertensive patients are informed about their disease, they did not change their lifestyle behavior^14,20^. So far, a little number of studies have compared the lifestyle behavior among aware and not aware hypertensive patients, which could be an important step for further treatments^20,21^. Therefore, the objective of the current study was to compare the healthy lifestyle behaviors among those who were and not aware of their hypertension. We additionally evaluate the percentages of controlled hypertension among those were aware of the disease.

## Results

A total of 2577 patients, with a mean age of 40.80 years (95% confidence interval: 40.60–41.01), were enrolled into the study. Baseline characteristics of participants were shown in Table 1. Of all patients, 49.83% were female. Those who were aware of their disease were older and less educated. Moreover, 56.10% of patients who were aware of their illness and 40.91% of unaware patients were male (p-value < 0.0001).

**Table 1:** The characteristics of hypertensive patients who were and were not aware of their hypertension status.

	Not aware (n=1213)		Aware (n=1364)		P-value	Total (n=2577)	
	%	95% CI	%	95% CI		%	95% CI
Sex							
Males	56.10	53.64–58.55	40.91	37.03–44.79	<0.0001	50.16	46.21–54.11
Females	43.90	41.44–46.35	59.09	55.21–62.96		49.83	45.88–53.78
Age (year)							
25–34	46.31	43.54–49.06	28.98	23.81–34.15	<0.0001	39.54	36.41–42.67
35–44	28.97	27.11–30.81	22.60	19.77–25.42		26.48	23.23–29.73
45–54	16.00	14.74–17.25	23.94	21.65–26.23		19.10	16.50–21.71
55–65	8.73	6.88–9.15	24.47	21.99–26.04		14.88	11.21–18.55
Residence							
Urban	72.96	70.66–75.26	75.75	72.15–79.33	0.350	74.05	72.01–76.09
Rural	27.04	24.73–29.33	24.25	20.66–27.84		25.95	22.91–22.50
Education							
Lower than diploma	58.88	54.34–63.41	70.59	64.80–76.38	<0.0001	63.45	59.15–67.75
Diploma	23.63	19.60–27.66	18.78	14.96–22.58		21.74	18.62–24.85
Higher than diploma	17.49	13.61–21.35	10.63	7.26–13.99		14.81	11.90–17.71
Antihypertensive medication use							
	—	—	74.51	73.13–76.78		47.85	42.11–53.58
	**Mean**	**95% CI**	**Mean**	**95% CI**		**Mean**	**95% CI**
Systolic blood pressure, (mm Hg)	139.08	137.03–141.13	136.40	134.35–138.46	0.007	138.03	136.17–139.91
Diastolic blood pressure (mm Hg)	92.53	91.52–93.54	85.01	83.66–86.35	<0.0001	89.59	88.51–90.66

CI; confidence interval. Values are reported as weighted percentage and weighted mean (data were weighted based on the 2012 national Iranian population aged ≥25 and ≤65 years old).

As presented in Tables 2, 27.79% of people who were not aware of their disease and 29.24% of people who had already been informed of their illness were adherence to the good lifestyle group. However, this difference was not statistically significant (p-value = 0.885). The results show that aware patients were more adherences to healthier lifestyle than those who were not aware. However, this significance finding is not true for physical activity.

**Table 2:** Adherence to healthy behaviors among hypertensive subjects by awareness of hypertension status*.

Adherence to healthy behaviors	Not aware (n=1213)		Aware (n=1364)		P-value	Total (n=2577)	
	%	95% CI	%	95% CI		%	95% CI
Fruits							
Poor	44.79	38.76–50.82	46.24	40.90–51.56	0.603	45.36	40.74–49.96
Moderate	19.31	16.25–22.36	20.72	16.91–24.53		19.86	17.55–22.17
Good	35.90	30.69–41.09	33.04	28.09–37.97		34.78	30.45–39.11
Vegetables							
Poor	51.72	45.66–57.77	51.45	44.71–58.19	0.581	51.62	46.17–57.05
Moderate	22.63	19.24–26.01	20.38	16.29–24.46		21.74	19.16–24.33
Good	25.65	19.83–31.47	28.17	21.71–34.63		26.64	21.26–32.01
Dairy products							
Poor	46.90	40.25–53.55	47.53	41.54–53.51	0.801	47.15	41.33–52.95
Moderate	30.30	25.67–34.91	31.27	26.58–35.96		30.68	27.01–34.34
Good	22.80	16.80–28.79	21.20	16.50–25.88		22.17	17.29–27.06
Fast-food							
Poor	23.38	19.34–27.42	22.01	18.10–25.92	0.905	22.84	19.50–26.19
Moderate	16.98	14.14–19.82	21.74	18.49–24.94		18.84	16.70–20.98
Good	59.63	54.13–65.13	56.24	51.80–60.68		58.31	54.41–62.21
Sweet soft drinks							
Poor	41.79	37.56–46.03	41.06	36.59–45.54	0.298	41.51	38.29–44.73
Moderate	25.19	22.12–28.27	27.33	23.40–31.26		26.03	23.38–28.67
Good	33.01	28.19–37.82	31.59	27.73–35.44		32.45	29.08–35.82
Unsaturated oil							
Poor	41.11	34.75–47.46	39.76	32.79–46.72	0.653	40.58	34.66–46.50
Good	58.89	52.53–65.24	60.24	53.27–67.21		59.41	53.49–65.33
Salt intake							
Poor	56.92	50.83–63.01	43.89	37.62–50.15	0.005	51.83	46.53–57.12
Good	43.08	36.99–49.16	56.11	49.84–62.37		48.17	42.87–53.46
Physical activity							
Poor	31.77	26.24–37.29	38.46	31.54–45.37	0.016	34.38	28.81–39.94
Moderate	31.21	26.80–35.61	30.92	25.27–36.56		31.10	26.98–35.21
Good	37.03	32.13–41.91	30.62	26.92–34.30		34.52	30.79–38.25
Smoking							
Smoker	14.20	10.98–17.41	10.03	7.43–12.62	0.043	12.57	10.37–14.77
Non-smoker	85.80	82.58–89.01	89.97	87.37–92.56		87.43	85.22–89.30
BMI							
Poor	70.96	66.42–75.48	75.41	71.08–79.74	0.166	72.70	69.53–75.86
Good	29.04	24.51–33.57	24.59	20.25–28.92		27.30	24.13–30.46
Total lifestyle score							
Poor	44.22	38.03–50.41	43.91	38.40–49.41	0.885	44.10	38.92–49.28
Moderate	27.77	23.32–32.23	26.85	23.11–30.58		27.42	24.11–30.72
Good	27.79	22.35–33.64	29.24	23.62–34.86		28.48	23.74–33.22

CI; confidence interval. Values are reported as weighted percentage (Data were weighted based on the 2011 Iranian census). *Fruits (poor: eating no fruit per day, moderate: 1 serving unit per day, good: more than 2 serving unit per day); vegetables (poor: eating no vegetables per day, moderate: 1 serving unit per day, good: more than 2 serving unit per day); dairy products (poor: drinking ≤ 1 unit per day, moderate: drinking 2 units per day, good: drinking ≥ 3 units per day); fast food (poor: eating ≥ 2 days a week, moderate: eating 1 day a week, good: eating no fast food per week); soft drinks (poor: ≥ 2 days a week, moderate: drinking 1 day a week, good: no drinking per week); unsaturated oil (binary covariate, poor: no, good: yes); salt intake (poor: using salt with daily food, good: not using with daily food); physical activity (poor: < 30 minute per week, moderate: 30–180 minute per week, good: more than 180 minute per week); smoking (poor: smoker, good: non-smoker); BMI (poor: overweight, good: normal weight).

There was a significant difference between those who were aware without medication and who were aware with medication regarding adherence to healthy lifestyle categories (Table 3). People who were aware without medication, were more tending to good lifestyle compared with those who were aware of medication (30.52% vs. 27.14%; p-value = 0.033). Additionally, we found that there is no significant difference in case of controlling hypertension and adherence to healthy lifestyle (p-value = 0.091) in the total population. However, in those without medication used, those who were able to control hypertension, adherence to good lifestyle category (32.54% vs. 27.59%; p-value = 0.042).

**Table 3:** Lifestyle behaviors among patients who were aware of their hypertension status and the status of controlling hypertension*.

		Total (n=1364)		Poor (n=597)		Moderate (n=374)		Good (n=393)		p-value
		%	95% CI	%	95% CI	%	95% CI	%	95% CI	
Total (n=1364)	Aware without medication use	46.06	40.82–51.29	41.80	33.82–49.77	27.67	22.18–33.16	30.52	21.61–39.40	0.033
	Aware with medication use	53.94	48.71–59.17	46.71	40.19–51.23	26.13	22.11–30.17	27.14	23.30–32.99	
Total (n=1364)	Controlled	50.47	44.91–56.03	39.99	32.63–47.36	30.00	24.07–35.93	30.01	21.40–38.59	0.091
	Uncontrolled	49.53	43.96–55.08	47.90	41.09–54.70	23.63	19.65–27.61	28.47	21.99–34.94	
				(n=169)		(n=118)		(n=115)		
Aware without medication use (n=402)	Controlled	59.25	51.48–67.03	35.83	25.69–45.96	31.63	22.94–40.31	32.54	19.99–45.15	0.042
	Uncontrolled	40.74	32.97–48.51	50.48	39.52–61.44	21.93	14.92–28.93	27.59	17.06–38.12	
				(n=428)		(n=256)		(n=278)		
Aware with medication use (n=962)	Controlled	42.98	35.74–50.21	44.91	37.11–52.69	28.08	20.99–35.16	27.01	20.46–33.55	0.217
	Uncontrolled	57.02	49.78–64.25	47.32	38.75–53.88	24.67	19.85–29.49	28.01	22.47–35.52	

CI; confidence interval. Values are reported as weighted percentage (Data were weighted based on the 2011 national Iranian census). *Fruits (poor: eating no fruit per day, moderate: 1 serving unit per day, good: more than 2 serving unit per day); vegetables (poor: eating no vegetables per day, moderate: 1 serving unit per day, good: more than 2 serving unit per day); dairy products (poor: drinking ≤ 1 unit per day, moderate: drinking 2 units per day, good: drinking ≥ 3 units per day); fast food (poor: eating ≥ 2 days a week, moderate: eating 1 day a week, good: eating no fast food per week); soft drinks (poor: ≥ 2 days a week, moderate: drinking 1 day a week, good: no drinking per week); unsaturated oil (binary covariate, poor: no, good: yes); salt intake (poor: using salt with daily food, good: not using with daily food); physical activity (poor: < 30 minute per week, moderate: 30–180 minute per week, good: more than 180 minute per week); smoking (poor: smoker, good: non-smoker); BMI (poor: overweight, good: normal weight).

Table 4 presents the results of multivariate logistic regression model for un-controlling hypertension in those who were aware of their hypertension. In total population the risk of un-controlled hypertension in people who adhere to moderate lifestyle was approximately 37% less than that in people with an unhealthy lifestyle (p-value = 0.01). Additionally, in those who were aware but did not use any antihypertensive medication, the odds ratio of adherence to moderate lifestyle was 0.39 (0.21–0.73); however, such a relationship was not observed in people who were adherence to good lifestyle. No significant associations were observed in those who were aware and used antihypertensive medications.

**Table 4:** Regression coefficients and 95% confidence intervals describing the association between lifestyle behaviors with uncontrolled hypertension among those who were aware of their hypertension status.

	Healthy lifestyle behaviors	Model 1			Model 2		
		OR	95% CI	P-value	OR	95% CI	P-value
Total (n = 1364)	Poor	Reference			Reference		
	Moderate	0.67	0.026	0.45–0.94	0.63	0.43–0.89	0.01
	Good	0.79	0.384	0.46–1.34	0.81	0.49–1.31	0.378
Without medication use (n = 402)	Poor	Reference			Reference		
	Moderate	0.49	0.38–1.42	0.024	0.39	0.21–0.73	0.004
	Good	0.60	0.26–1.16	0.102	0.55	0.23–1.12	0.081
With medication use (n = 962)	Poor	1			1		
	Moderate	0.85	0.54–1.26	0.410	0.89	0.48–1.30	0.332
	Good	1.04	0.63–1.71	0.822	0.99	0.59–1.64	0.873

CI; confidence interval. Model 1: crude odds ratio, Model 2: adjusted for age, sex, education, antihypertensive medication (only for total population). Data were weighted based on the 2011 national Iranian census.

Adherence to healthy lifestyle behaviors among those who were aware of hypertension by controlling hypertension situation were presented in Supplementary Table 1.

## Discussion

The present study assessed adherence to the healthy lifestyle in different subgroups hypertensive patients. Based on the official guidelines on hypertension treatment, lifestyle modification is the first line of treatment for hypertensive patients^14^, and it is expected that people’s awareness of hypertension result in changes in people’s lifestyles. However, the results of our study showed no significant difference in lifestyle between people with hypertension who were aware of their illness and those who were not aware of their illness. Nevertheless, it is expected that aware patients modify their lifestyle, or at least behave in a way different from those who are not aware of their illness.

The only significant difference between the two groups was in terms of salt consumption and cigarette smoking. There are some studies reporting that the awareness of hypertension can affect people’s lifestyle and results in changes^21–23^, while there are some other studies indicating no significant difference in lifestyle between the two groups of patients; those who are aware and those who are unaware of their illness^20^. As stated above, some studies have reported changes in lifestyles after being informed of the disease; this finding may be attributed to a number of reasons. First, the difference observed in many articles is very small; on the other hand, a large number of these articles have reached such a conclusion through questioning people about the modification of their lifestyle. Nevertheless, some studies have shown that patients usually overestimate the modification of their lifestyle^22^. the results of this study indicate that awareness of the disease resulted in a reduction in salt intake and cigarettes smoking, but did not affect other lifestyle risk factors. Our results are in line with the results of other studies conducted in European countries. Based on the results of such studies, there is a significant difference between those aware of their illness and those unaware of their illness in terms of salt and alcohol consumption and smoking, but there is no significant difference between the two groups in terms of other lifestyle risk factors^21,24^. In a study conducted in Korea and Spain, it was found that people who were aware of their hypertension, as compared with those who were not aware of their illness, consumed less salt and smoked less frequently^20,24^. This is probably due to the extensive trainings on the effect of these two variables on hypertension.

There are a few justifications for the lack of change in lifestyle and the lack of significance difference in lifestyles between those aware of their illness and those unaware of their disease. As the first justification, there are not adequate and scientific trainings for those whose disease is diagnosed^21^. Aparently, there are broad and extensive trainings for patients to reduce the consumption of salt, but there are not enough training about the consumption of fruits and vegetables and other lifestyle risk factors. On the other hand, patients with hypertension seem to assume that taking antihypertensive drugs is enough to control the disease and thus they do not feel the need for lifestyle modification^20^. Finally, lifestyle modification is difficult and adherence to a healthy lifestyle is not an easy task, as it requires appropriate trainings and interventions.

The lack of a relationship between lifestyle and hypertension control in people who are taking medication may be due to different reasons. For instance, people who are taking medication are likely to have a higher level of hypertension than those who do not take medication; in the earlier group, lifestyle modification may not have a remarkable effect on hypertension control and they may still need to take medications. Moreover, uncontrolled hypertension in people taking drugs may be due to irregular use of medications. However, as we do not have any information about the medication compliance, we cannot make any conclusion in this area.

It is worth noting that people who were not aware of their disease had higher levels of physical activity, as compared with those who were aware of their disease. As a reason, it might be related to age. People who were not aware of their disease were younger and they were more likely to have a higher level of physical activity than older people with hypertension. Moreover, health policymakers should pay attention to the fact that people who were not aware of their illness had a lower mean age than other patients, indicating the need for more frequent screening the disease in younger people.

As an interesting point in the present study, we observed different results when we limited our analysis to people who were aware of their illness and divided them into two groups of patients who were taking antihypertensive drugs and those who did not take any medication. Indeed, there was a significant relationship between lifestyle and taking antihypertensive drugs and their effect on hypertension control. People who were aware of their illness and did not take medications had a better lifestyle than those who had hypertension and take antihypertensive medication. On the other hand, hypertension control in people who did not use any drug had a significant relationship with ahealthy lifestyle, while such a relationship was not observed in people taking medications. Nonetheless, a large percentage of this group of patients had uncontrolled hypertension. This may be due to two reasons. First, the lack of such a relationship in people who were taking antihypertensive drugs is not due to the absence of a relationship but is rather due to the lack of adequate difference in lifestyle between the two groups with controlled and uncontrolled hypertension. Second, as noted above, patients who are taking antihypertensive drugs do not feel a need to modify their lifestyle. Some studies have shown that the use of antihypertensive drugs increases the odds ratio of non-adherence to a healthy lifestyle, which can be a justification for our finding^25,26^. It is clear that a large number of patients do not have a healthy lifestyle, and hypertension is not controlled in a large number of them, although they are aware of their illness. Therefore, health professionals and physicians must put more emphasis on lifestyle modifications when counseling patients or managing hypertension.

As one of the most important strengths of the present study, for the first time, we used the data collected at the national level. Thus, our study assessed lifestyle and hypertension control among a population that is representative of all hypertensive patients in the country.

As one of the limitations of our study, we did not access any specific data, for example the data on marital status, socioeconomic status, stress, consumption of red meat, or drug compliance; these may affect the results of the study, as we were unable to measure them.

We also did not access the data on two variables, including the amount of salt intake and oil consumption. Therefore, we used the two variables of salted food intake and the type of oil used, as proxies for the use of these two items.

## Conclusion

The results of the present study showed that lifestyle of people who are aware of their illness is not different from the lifestyle of those who are not aware of their illness. However, of people who are aware of their illness, people taking antihypertensive drugs are significantly different from those who do not take any medication in terms of the relationship between lifestyle and hypertension control.

In people who do not use any antihypertensive drug, healthy lifestyle showed a significant relationship with hypertension control. However, such a relationship was not observed in people who were taking antihypertensive drug. People who take medicine seem to be sure about the control of their hypertension and do not feel any need to modify their lifestyle. However, a large percentage of these people do not have controlled hypertension, and their lifestyle may be a reason for not controlling hypertension in a large number of them. Therefore, apparently one of the most important factors in controlling hypertension is to take antihypertensive drugs concurrent with lifestyle modification. Health policymakers must pay attention to this important item and implement effective relevant interventions.

## Materials and Methods

### Study population

**Figure 1:**
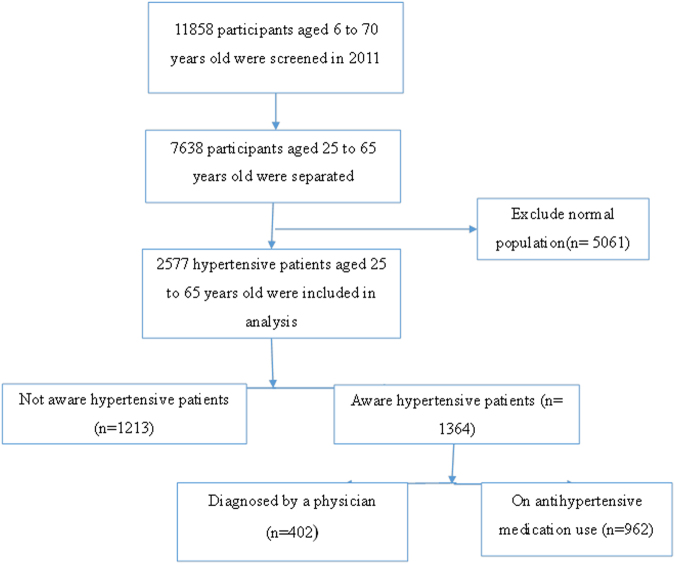
Flowchart of the participant selection.

The present research was conducted using the data collected by the national study of major risk factors for non-communicable diseases (SuRFNCD) of Iran, in 2011. Details on this study are reported elsewhere^13,27^. In brief, the mentioned study was conducted on 11858 people aged 6 to 70 years old, based on a step-wise approach recommended by the World Health Organization. The samples were selected using stratified cluster sampling method (provinces, cities, or villages were selected as strata) and the clusters were selected from each stratum. Only people who were aged 25 to 65 years old and had hypertension were considered into this study (Fig. 1).

### Measures of exposures

Height and weight were measured for each individual without shoes and in light clothing. Standard questionnaire based on the World Health Organization’s guidelines for the step-wise survey of risk factors for non-communicable diseases were used to collect demographic information including age, sex, education, and occupation, as well as lifestyle related behaviors such as nutrition (seven variables), physical activity (the Global Physical Activity Questionnaire)^28^, and cigarette smoking.

In order to evaluate participants nutritional status, seven variables, including the consumption of fruits, vegetables, dairy products, fast food, soft drinks, salt intake, and the type of consumed oil were considered. Two questions were used to measure the nutritional score on consumption of fruits, vegetables, and dairy products. In the first question, they asked about the number of days that one person consumes fruits, vegetables, or dairy products, and the second question was used to collect information on the mean number of consumed items per day. Using these two question, the mean number of items consumed per day was calculated. The number of days per week based on fast food and soft drinks (sweet drinks) were also recorded^29^.

In the present study, salt intake was evaluated by this question “are you using salt with your meal?”. Using the related questionnaire, the type of using oil (saturated and unsaturated oil) was recorded. Smoking status was evaluated by the current question “are you currently smoking any kind of cigarette (factory manufactured cigarettes, handcrafted cigarettes, or cigars)?”

Self-reported hypertension medication was considered as using antihypertensive medication^13^. Blood pressure was measured twice on the seating position on their right arm with five minute distance between two measurements. The mean value of both systolic blood pressure (SBP) and diastolic blood pressure (DBP) were considered as person’s blood pressure measurements.

### Definition of outcomes

Hypertension was defined as SBP ≥140 mm Hg or DBP ≥90 mm Hg or using any antihypertensive medication or had been diagnosed as hypertensive cases by physician^24^. Participants who were used medication or had been diagnosed as hypertensive cases by physician categorized into aware group. While, those who were not aware of their hypertension situation, were categorized as unaware patients. Additionally, we defined controlled hypertensive cases as those who were aware and his/her SBP and DBP were <140 mm Hg and <90 mmHg, respectively^24^.

### Method of scoring the lifestyle variables

A lifestyle score was computed for each participant based on their total nutritional status scores (fruits, vegetables, dairy drinks, fast food, soft drinks, salt intake, type of oil), physical activity, obesity and smoking scores. The lifestyle score of every individual was calculated through summing up the total scores of named factors, and the lower scores showed that the subject had an unhealthy lifestyle.

Table 5 show the tertile cut offs for scoring of each lifestyle variables. To determine the nutrition score, first, we defined tertiles for each variable, and the subjects with a bad (poor status) to good (fair status) nutrition status were given a score of 1 (for the first tertile) to 3 (for the third tertile). The variables such as fast food or sweet drinks consumption were scored reversely^30^.

**Table 5:** Definition of healthy lifestyle scoring.

Lifestyle factor	Criteria	Score according tertile cut offs
Fruit	Number of units per day	Eating no fruit per day = 1 Eating 1 serving unit per day = 2 Eating more than 2 serving unit per day = 3
Vegetables	Number of units per day	Eating no vegetables per day = 1 Eating 1 serving unit per day = 2 Eating more than 2 serving unit per day = 3
Dairy products	Number of units per day	Drinking ≤1 unit per day = 1 Drinking 2 units per day = 2 Drinking ≥3 units per day = 3
Fast food	Number of days per week	Eating ≥2 days a week = 1 Eating 1 day a week = 2 Eating no fast food per week = 3
Soft drinks	Number of days per week	Drinking ≥2 days a week = 1 Drinking 1 day a week = 2 No drinking per week = 3
Oil	Saturated/Unsaturated	Consumed unsaturated oil = 1 Consumed saturated oil = 0
Salt intake	Yes/No	Not using salt with daily food = 1 Using salt with daily food = 0
Overweight	Yes/No	Normal weight (BMI; between 18.5 and 25) = 1 BMI ≥25 = 0
Smoking	Yes/No	Non-smoker = 1 Current smoker = 0
Physical activity	The times spent on recreational physical activity or walking	Lower than 30 minutes per week = 0 30–180 minute per week = 1 More than 180 minutes per week = 2

The times spent on recreational physical activity or walking were summed up. At the end, after defining tertiles for each variable, the subjects were categorized into three groups with poor, moderate, and good physical activity with a score of one to three, respectively.

People who did not consume salt received a score of one and the rest received a score of zero. Those who consumed unsaturated oil, did not smoke, and had a normal weight (Body mass index between 18.5 and 25) received a score of one for each variable and the other participants received a score of zero^31^. Body mass index (BMI) was calculated as weight divided by height squared (kg/m^2^).

At the end, all the scores were summed up and the final scores, based on the tertile system, were again categorized into three groups, bad, moderate and good lifestyle for first, seconed and third tertile, respectively^32^.

As the prevalence of alcohol consumption was very low, it was not included in determining lifestyle score.

### Statistical analysis

The weighted means (95% CI) and the weighted percentage (95% CI) were used to describe continues and categorical data, respectively based on survey analysis (weighted based on 2011 Iranian census based on gender and age categories). Student t test and Chi-square test were used to compare the two groups in terms of mean values and frequencies, respectively.

Since about 4.51% of covariates were missed, single imputation method was used based on regression model for estimation of missing information by using mice package in R software.

**Figure 2:**
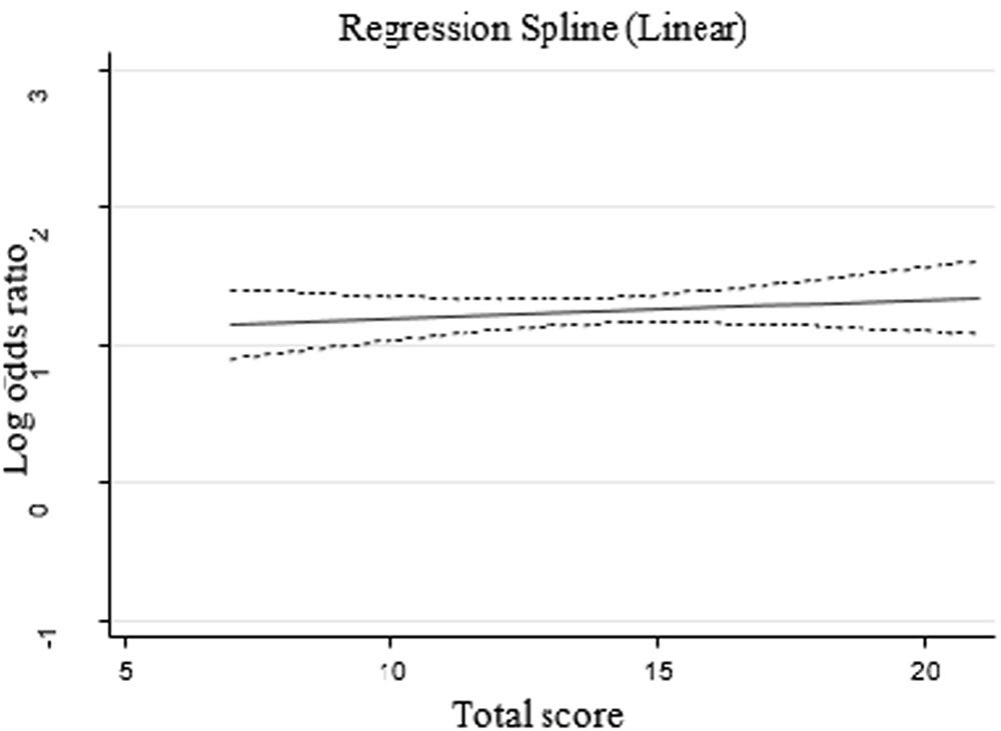
Cubic spline of lifestyle total score and control of hypertension.

Restricted cubic spline was used to investigate the continues association between total lifestyle score and control of blood pressure^33,34^. Knots were defined at the 25%, 50% and 75%. Since the linear association was detected, dichotomous healthy lifestyle variable was defined, with a poor, moderate and good category based on the total score tertiles (Fig. 2).

Multivariate Logistic regression model was used to determine the relationship between lifestyle modifications and un-control hypertension in people who had previously been aware of their illness. the interaction between lifestyle categories and antihypertensive medication was check and since it was significant (p-value < 0.05), analysis was done separately for those with and without medication as well as total population.

### Ethical approval

For all the procedures performed in SuRFNCD study, verbal informed consent was obtained from all individual participants in the study.

## Electronic supplementary material

Supplementary Table 1
