# Supplementary material for: Healthy lifestyle behaviors and control of hypertension among adult hypertensive patients
Source: Sci Rep. 2018 May 31;8:8508. doi: 10.1038/s41598-018-26823-5 (PMC5981436; doi:10.1038/s41598-018-26823-5)
Supplement: Supplementary file 1 — Supplementary Table 1 [file 41598_2018_26823_MOESM1_ESM.pdf]

Title:

Healthy lifestyle behaviors and control of hypertension among adult hypertensive patients

Samaneh Akbarpour[1]; Ph.D, Davood Khalili[2]; MD, Ph.D, Hojjat Zeraati[1]; Ph.D, Mohamadali Mansournia[1]; MD, Ph.D, Azra Ramezankhani[2];Ph.D, Akbar Fotouhi[1*]; MD, Ph.D

[1] Department of Epidemiology and Biostatistics, School of Public Health, Tehran University of Medical Sciences, Tehran, Iran.

[2] Prevention of Metabolic Disorders Research Center, Research Institute for Endocrine Sciences, Shahid Beheshti University of Medical Sciences, Tehran.

Corresponding author: Dr Akbar Fotouhi; Department of Epidemiology and Biostatistics, School of Public Health, Tehran University of Medical Sciences, Tehran, Iran, **Email:** afotouhi@sina.tums.ac.ir

Supplementary Table 1. Adherence to healthy behaviors among hypertensive subjects by awareness of hypertension status*

	**Adherence to healthy behaviors**	**On medication use (n=962)**		**P-value**	**Without medication use (n=402)**		**P-value**
		Controlled (n=379) Percent (95% CI)	Uncontrolled (n=583) Percent (95% CI)		Controlled (n=195) Percent (95% CI)	Uncontrolled (n=207) Percent (95% CI)	
Fruits	Poor	44.32 (34.82-53.82)	45.38(38.97-51.78)	0.394	43.08(33.46-52.69)	54.60(43.86-65.33)	0.032
	Moderate	21.94(15.07-28.79)	21.72(17.02-26.43)		23.03(14.43-31.62)	16.23(9.92-20.54)	
	Good	33.74(25.80-41.67)	32.90(26.26-39.53)		33.89(22.80-44.96)	29.16(22.05-38.26)	
Vegetables	Poor	46.26(36.89-55.62)	47.52(39.90-55.13)	0.644	54.98(45.93-72.03)	56.27(45.45-67.08)	0.083
	Moderate	21.91(15.52-28.30)	21.62(16.03-27.22)		18.16(10.99-24.99)	22.56(12.55-32.57)	
	Good	31.83(24.15-39.49)	30.85(23.42-38.27)		26.85(16.92-36.78)	21.16(12.55-32.57)	
	Poor	48.34(39.28-57.39)	49.34(41.87-56.81)	0.133	42.13(30.90-53.36)	51.41(41.67-61.14)	0.098

Dairy products	Moderate	29.34(21.81-36.88)	29.20(23.13-35.26)		33.81(23.88-43.74)	33.36(23.48-43.23)	
	Good	22.31(15.08-29.53)	21.46(15.52-27.39)		24.05(14.97-33.13)	15.22(8.56-21.88)	
Fast-food	Poor	22.04(16.51-27.56)	27.30(20.11-34.48)	0.001	22.1(12.69-31.62)	15.23(7.06-23.40)	0.001
	Moderate	16.94(13.34-20.52)	20.72(15.21-26.22)		26.54(19.09-33.99)	23.91(15.32-32.48)	
	Good	61.03(55.06-66.98)	51.98(45.08-58.88)		51.28(41.01-61.57)	60.86(49.04-73.67)	
Sweet soft drinks	Poor	34.86(28.36-41.34)	37.41(31.32-43.48)	0.261	49.93(38.79-61.06)	41.85(30.79-52.92)	0.220
	Moderate	27.82(21.72-33.93)	25.51(20.73-30.26)		26.85(17.23-36.47)	30.45(22.38-38.52)	
	Good	37.32(28.75-45.87)	37.09(30.47-43.71)		23.22(16.03-30.39)	27.69(18.57-36.81)	
Unsaturated oil	Poor	32.64(22.42-42.86)	42.72(34.83-50.59)	0.051	43.67(31.63-53.71)	39.45(27.47-51.44)	0.679
	Good	67.35(57.13-77.57)	57.28(49.40-65.16)		58.33(46.28-68.36)	60.54(48.55-72.52)	
Salt intake	Poor	37.48(29.69-45.26)	49.67(42.61-56.72)	0.042	33.76(21.47-46.05)	42.51(29.15-55.86)	0.025
	Good	62.5154.73-70.30)	50.33(43.27-57.38)		66.23(53.94-78.52)	57.49(44.13-70.84)	
	Poor	41.53(33.63-55.43)	39.30(31.84-46.76)	0.544	37.82(25.94-49.70)	30.50(21.37-39.62)	0.484

Physical activity	Moderate	26.24(17.66-34.82)	26.98(21.46-32.51)		38.62(26.70-50.54)	34.04(24.01-44.08)	
	Good	32.22(24.43-38.07)	33.71(27.70-39.71)		30.87(20.24-41.50)	28.13(19.80-36.46)	
Smoking	Poor	10.93(6.88-14.96)	8.98(5.04-12.93)	0.205	9.02(3.02-15.01)	12.09(5.05-19.12)	0.764
	Good	89.07(84.03-94.11)	91.01(87.06-94.95)		90.98(84.98-96.97)	87.91(79.87-95.94)	
BMI	Poor	79.73(73.60-85.86)	77.31(72.10-82.52)	0.839	66.87(54.33-79.39)	79.40(70.11-88.71)	0.036
	Good	20.27(14.14-26.39)	22.69(17.48-27.89)		33.13(20.60-45.66)	20.59(11.29-29.89)	

CI; confidence interval

values are reported as weighted percentage (Data were weighted based on the 2011 national Iranian census).

*fruits (poor: eating no fruit per day, moderate: 1 serving unit per day, good: more than 2 serving unit per day); vegetables (poor: eating no fruit per day, moderate: 1 serving unit per day, good: more than 2 serving unit per day); dairy products (poor: drinking ≥1 unit per day, moderate: drinking 2 units per day, good: drinking ≤3 units per day); fast food (poor: eating ≥2 days a week, moderate: eating 1 day a week, good: eating no fast food per week); soft drinks (poor: ≥2 days a week, moderate: eating 1 day a week, good: eating no fast food per week); unsaturated oil (binary covariate, poor: no, good: yes); salt intake (poor: using salt with daily food, good: not using with daily food) ; physical activity (poor: < 30 minute per week, moderate: 30-180 minute per week, good: more than 180 minute per week); smoking (poor: smoker, good: non-smoker); BMI (poor: obese, good: non-obese).
